# Impact of patient aggression and violence against physicians on the team and organisational levels in China: a qualitative study

**DOI:** 10.1136/bmjopen-2024-092229

**Published:** 2025-05-28

**Authors:** Yuhan Wu, Kees Ahaus, Dahai Zhao, Martina Buljac-Samardzic

**Affiliations:** 1 Erasmus School of Health Policy & Management, Erasmus University Rotterdam, Rotterdam, The Netherlands; 2 School of International and Public Affairs, Shanghai Jiao Tong University, Shanghai, China

**Keywords:** Hospitals, Physicians, Work Satisfaction, Health Workforce

## Abstract

**Abstract:**

**Objectives:**

Aggression and violence against physicians in hospitals is acknowledged to be an issue, and patients (and their relatives/friends) have been identified as the most prevalent source. The aim of this study is to investigate the impact of patient aggression and violence against physicians on the team and organisational levels.

**Design:**

This is a qualitative interview study based on semistructured, in-depth individual interviews. Interview transcripts were coded and analysed in Atlas.ti.

**Setting:**

Interviews were conducted in Chinese hospitals.

**Participants:**

This study involved 29 diverse participants, including physicians, hospital team leaders and hospital board members, working in two secondary hospitals and two tertiary hospitals in China.

**Results:**

This study found that, at the team level, aggression and violence by patients (and their relatives/friends) can affect team climate, team communication, team beliefs and team resources. At the organisational level, such aggression and violence can have negative financial impacts (ie, involving compensation and additional costs) and societal impacts (ie, image and reputational damage, and public distrust). Although peer support and leaders’ support were identified as important sources for physicians to deal with violent incidents, these sources were not used to their full potential.

**Conclusions:**

Recovering a team climate after a violent incident and providing diverse forms of support, especially proactive support from leaders and peers, represent two important approaches to cope with the negative impact of patient (and their relatives/friends) aggression and violence against physicians on both team and organisational levels.

**Strengths and limitations of this study**

- Data were collected from physicians, team leaders and hospital board members, which provided a comprehensive perspective of the impact of patient aggression and violence on teams and organisations.
- Although participants were recruited through the authors’ networks, the potential for selection bias was minimised by including individuals from various regions, hospital levels and roles.
- This study was conducted in China, a research setting where there is extensive experience with patient aggression and violence against physicians and therefore a wide understanding of the consequences from physicians. Although these findings offer valuable insights for other settings, this may limit the generalisability of the findings to other countries.

## Introduction

 Aggression and violence in hospitals is acknowledged as an issue that arouses concern in society.^1^ Among healthcare professionals, physicians are at a high risk of experiencing aggression and violence in hospitals.^2^ Although workplace violence in hospitals can come from both internal sources (eg, colleagues and leaders) and external sources (patients and their relatives/friends), the latter group has been identified as by far the most prevalent source.^3 4^

The consequences of patient (and their relatives/friends) aggression and violence against physicians are widely acknowledged in practice and in the literature.^24^,^7^ Although, these consequences have been studied from multiple angles and on different levels, most studies have focused on the individual level. More specifically, physicians who had experienced patient violence and aggression mostly reported consequences with a psychological (eg, depression), emotional (eg, anger and fear) and/or work-functioning character, such as reduced job satisfaction.^5 8^ Such aggression and violence also impact team functioning and have consequences for the hospital. For example, violence in the workplace can make team members feel unsafe, which affects the group climate and the establishment of team relationships at work.^9^ Further, the team climate can impact a team’s behaviour and productivity, such as its performance, creativity, cooperation and its employees’ turnover intention.^10^,^14^ Organisational-level consequences could include reduced performance and compensation costs due to patient violence, leading to physicians taking time off work due to injury.^4 8^ Overall, the impact of patient aggression and violence against physicians has been relatively poorly researched at the team and organisation levels compared with the consequences on the individual level, and the potential consequences warrant a more comprehensive investigation.

A wide range of worldwide studies have highlighted the prevalence of patient aggression and violence and indicated its negative impact on healthcare professionals and institutions. In Europe, physicians in Bulgaria and Spain frequently experience violence from patients and their families, resulting in psychological and work-functional impacts.^15 16^ Belgian and Italian studies have shown severe risk factors associated with patient aggression and violence against healthcare workers, both at the individual and at the systemic level.^17 18^ Multiple studies in Turkey and India have shown that the prevalence of workplace violence against physicians frequently occurs in emergency departments, leading to occupational stress and burnout.^19^,^21^

Although aggression and violence in healthcare settings is a global issue, its prevalence and impact vary by culture and healthcare system.^22^ In China, more than 60% of healthcare professionals have experienced workplace violence, particularly from patients and their relatives.^23 24^ China’s healthcare system also faces persistent challenges that place significant strain on hospitals, increasing the risk of patient aggression and violence. These challenges include high patient expectations, resource limitations and a preference for patients to directly seek care at higher-level hospitals due to the lack of a primary care gatekeeper system.^25 26^ As such, Chinese hospitals form a research setting in which there is extensive experience with patient aggression and violence against physicians and therefore a wide understanding of the consequences from physicians not only as victims but also as witnesses of such incidents. Insights from this study will also be valuable for other hospitals in other countries that encounter less patient aggression and therefore lack the experience to fully understand the consequences and severe impacts of such incidents.

This study aims to understand the impact of patient (and their relatives/friends) aggression and violence against physicians on the team and organisational levels through semistructured interviews. Based on the findings, strategies to address the negative impacts of patient (and their relatives/friends) aggression and violence on the team and hospital levels can be developed.

## Methods

### Research design and participants

Given the limited number of studies that have investigated the effects of patient (and their relatives/friends) aggression and violence against physicians on the team and organisational levels, a qualitative study was appropriate to explore the impact on these levels. The interview design combined semistructured and open-ended questions (see online supplemental appendix 1 for the interview guidelines). To capture the consequences on the team and organisational levels, this study involved three types of people working in hospitals who are exposed in different ways to patient aggression and violence: (1) physicians who have experienced verbal and/or physical violence from patients (and/or their relatives/friends) or witnessed it against team members (ie, fellow physicians); (2) leaders of physician teams; and (3) hospital board members. The combination of these three groups provided a comprehensive view of the impact of patient (and their relatives/friends) aggression and violence on teams and organisations. To attain a diverse sample, four public hospitals (two secondary and two tertiary) located in eastern China (ie, Shanghai, Shandong, Jiangsu and Zhejiang) were selected. These regions were selected for the variety in hospital landscapes. Specifically, Shanghai possesses advanced medical technology and famous hospitals, which attracts many Chinese patients. In contrast, due to urban–rural divide, Shandong faces a range of problems (eg, distribution of healthcare resources) that impact access to healthcare services. With proactive government policies and a well-developed economy, Jiangsu has a well-organised hospital management and physician training programmes that set an example for improving healthcare efficiency. Zhejiang is known for the widely recognised hospital’s management model through healthcare reforms and an innovative service model of urban–rural healthcare integration (eg, digital healthcare). The regions present a comprehensive view of the hospital dynamics in Chinese hospitals.

Participants were recruited using the authors’ own networks. The intention was to achieve data saturation, and the required number of interviews depended on the nature of the topic, the quality of the data and the method of analysis.^27^ This study started by inviting, from each hospital, one board member, two team leaders and at least four physicians who were part of a monodisciplinary team with the same specialism. The number of participants was increased based on accessibility (additional board members were beyond the reach of this study) until data saturation was achieved. A total of 29 participants were interviewed between April and July 2023. The final sample contained 4 board members, 8 team leaders and 17 physicians. Of these participants, 16 were male. Detailed participant information is provided in online supplemental appendix 2.

### Data collection

This study collected data using semistructured interviews. To test whether the interview questions were understandable and appropriate, pilot interviews were first conducted. Five participants were interviewed in this pilot, including four physicians (from gynaecology and obstetrics, orthopaedics, nephrology and otolaryngology departments) and one team leader (from an ophthalmology department) from different Chinese hospitals. Based on the outcome of the pilot study, the interview questions were refined by tailoring them to the different types of respondents (ie, physicians, team leaders and board members).

Following the revisions, the physicians were primarily asked about the impact of patient (and their relatives/friends) aggression and violence on their daily teamwork. In addition, team leaders and board members were also asked about the impact of this type of aggression and violence on the hospitals and the role of leadership. All the interviews were conducted face-to-face in the Chinese hospitals that the respondents were affiliated with and were audio-recorded and transcribed. Interviews lasted between 25 and 65 min (average 32 min) and did not conflict with the participants’ work.

### Data analysis

The first author transcribed the interviews and anonymised them by assigning identification numbers (physicians: P1–P17; team leaders: T1–T8; and board members: B1–B4). These identifiers are used in the quotes included in this paper. The interviews were conducted in Chinese by the first author (YW), and a subset of the interviews was translated into English. The qualitative data were analysed using the method proposed by Gioia *et al*,^28^,^30^ including (1) generating first-order codes (ie, raw codes directly extracted from the interview transcripts); (2) integrating first-order codes into second-order themes through axial coding and (3) ultimately refining higher-order theoretical frameworks. On this basis, data coding and analysis were carried out using Atlas.ti, including the following sequential steps:

1. One author (YW) derived a comprehensive list of first-order codes (in English) based on all 29 transcripts. To assess the reliability of this coding, one external researcher (HW) independently examined two randomly selected transcripts and coded them against the full set of first-order codes.
2. The open codes were then combined, through axial coding, into second-order themes. Each researcher was involved in parts of the first-order codes, analysing their underlying meanings, and contributed to the development of second-order themes by interpreting the data through their perspectives and expertise. These second-order themes were then compared and any differences were discussed until a consensus was reached.
3. Finally, the second-order themes were grouped into integrative dimensions to form a structured and comprehensive framework for understanding the impact of patient aggression and violence at the team and organisational levels.

### Patient and public involvement statement

Patients and/or the public were not involved in this study.

## Results

### Team-level impacts

#### Team climate: unsafe and depressed

According to the respondents, patient (and their relatives/friends) aggression and violence against one of the physicians would have an immediate negative consequence for team climate, expressed by safety concerns, and consequently a depressed team atmosphere. The fear of physical harm or verbal abuse created a pervasive sense of a lack of safety among all team members, irrespective of whether they had directly encountered or witnessed such incidents. Respondents voiced concerns about potentially becoming the next victim of such aggression and violence given their similar working conditions: “*My colleague and I had similar job duties and content, and I was terrified that the same thing would happen to me*.” (P4) Moreover, physicians could encounter patient (and their relatives/friends) aggression and violence that was directed at them by chance—in response to a team member’s actions rather than their own:

Sometimes the violence was directed at the entire department. I mean, a patient’s frustration with one physician may be taken out on other physicians in the same department. It has also happened that a patient wanted to attack a specific physician but, when that physician was not in the office, such violence can be transferred to other members of the department. (B1)

The aftermath of raised safety concerns was a depressed atmosphere. This depressive team atmosphere could be traced back to team members empathising with each other and the effortless spread of individual negative emotions throughout the entire team:

We could understand how the victims felt (…) We were all sad. We treated our patients so well, but they did not understand us and even wanted to hurt us. (P5)

And

The victim was in a bad mood after experiencing such an incident. We were also easily influenced (…) I felt the whole atmosphere in the department became depressed. (P3)

As a consequence, under such circumstances, it was easy for team members to lose enthusiasm for their work, at least for a short period: “*I have noticed that team members were less motivated about their work than they used to be*.” (T3)

#### Decreased desire to communicate within teams

Respondents indicated that the occurrence of violence and aggression leads to a decline in communication within the team. This was predominantly observed in two dimensions: the communication between the victim and other team members, as well as communication within the entire team. The main reason for victims’ reluctance to communicate was based on their negative emotions: *“I did not want to communicate with others because I was in a bad mood.”* (P15) Victims also expressed that, for some time after, they found it challenging to concentrate on work due to safety concerns. The victim’s lack of focus was also noticed by team members in that they indicated that such circumstances interfere with effective conversations: *“I needed to question (the victim) several times to get a response. Sometimes the answers did not match the questions I asked.”* (P2)

Moreover, many respondents mentioned that even though they were not victims, communication within the whole team would be affected by a negative atmosphere: *“The whole atmosphere was negative, and we did not want to talk much.”* (P3) Respondents said that even talking about trivial matters seemed inappropriate: *“Normally we share funny things, but, in that situation, we certainly did not discuss them.”* (P17)

#### Team beliefs: better to offer low-risk treatment

There was an overall belief that it is better to be safe than sorry. Most respondents expressed the view that risk-reduction behaviours had become common within healthcare teams. Many respondents indicated that they increasingly opted for conservative treatment methods out of concern for their own safety and to avoid disputes. For instance, *“We like to opt for a more conservative treatment approach. For example, we avoid some high-risk treatments, even though this treatment might lead to better results in some cases.”* (T1) Respondents also reported that they tended to recommend patients to undergo a thorough medical examination, although some tests were not strictly necessary. At secondary hospitals, physicians were more likely to refer patients with complex conditions to other hospitals to protect themselves from potential violence and aggression, which is perceived as more likely during complex, high-risk and expensive medical procedures. This referral behaviour will affect the advancement of medical skills in the whole team: *“Sometimes, we opted for a referral, even though we could have cured the disease. This hinders physicians or the team learning new techniques.”* (T8)

#### Team resources: impacts on material and human resources

Patient (and their relatives/friends) aggression and violence has an impact on team resources in two main ways: damaging material resources and diminishing human resources. Respondents indicated that aggression and violence from patients (and their relatives/friends) can result in damage to material resources needed to treat patients:

Patients are likely to take out their frustration on some items. For example, they may break the office computer or some medical equipment (…) Some important medical equipment was smashed, and so we had no way to treat some patients quickly. (T7)

Team leaders reported that violent incidents may result in injuries or emotional distress to physicians, requiring adjustments to work schedules or the provision of leave to allow victims to recover. This may result in teams facing staff shortages in the short term:

We will let the victim take a few days off. Or sometimes we will ask a colleague to accompany the victim to the local police station to make a statement. This will affect the normal medical practice because of the shortage of people. (T3)

In the long term, team leaders noted that the occurrence of patient (and their relatives/friends) aggression and violence can create challenges in attracting and retaining employees, especially for high-risk departments (such as the emergency care):

Sometimes intern physicians will witness such events happening and they may then change their employment intentions. Sometimes new employees may also choose to leave or to join another, safer team when faced with such an incident. (T1)

#### Support from team members and leaders: valuable yet limited

Although the above findings suggest that victims are reluctant to engage in conversations, some would actively seek help. Seeking emotional support, such as verbal comfort from other team members, was perceived as a useful way to relieve negative emotions: *“When I experienced such violence, I liked to confide in my colleagues. After that, I would feel better. Of course, my colleagues will also take the initiative to care about me.”* (P9) The power of peer support was also reflected in the aftermath of a violent incident. Team members would take over the victim’s tasks during their absence. For example, “*Other team members will perform the operation in the victim’s place and briefly take over the care of their patients*.” (T4), and most team members did not perceive this type of support as an increased workload: *“I did not consider this situation as adding to my workload. We all help each other out. I may be in the same position 1 day.”* (P6)

Moreover, respondents indicated that the leader could play an important role since attention and support from leaders are likely to be more helpful for the victim in “coming out of the woods”. Team leaders could also reach out to hospital administrators (eg, board members) for assistance given their experience with dealing with such incidents:

They (hospital administrators) tend to have more experience and insights. I mean they may have experienced what you are experiencing, and they can tell you how to solve it based on their own experience. (T4)

Despite the recognition of the value of diverse support sources (from team members, team leaders, and hospital administrators), in practice, the support for victims is mainly singular in form, namely (verbal) support from their peers: *“The help we can get is limited and mostly verbal comfort. In fact, we would all like to have more forms of support, such as paid vacations.”* (P15)

### Hospital-level impacts: societal and financial impacts

The effects of patient (and their relatives/friends) aggression and violence at the hospital level had two main dimensions: societal and financial impacts. In terms of societal impact, aggression and violence by patients (and their relatives/friends) can damage the image and reputation of hospitals, potentially leading to public mistrust in them. Many respondents pointed out the negative role that the media can play. For example, some media reports may exaggerate the impact of an incident, resulting in reputational damage to the hospital.

In terms of financial impacts, the influence of aggression and violence by patients (and their relatives/friends) was concentrated in three areas. First, such aggression and violence can involve legal payments and costs related to the required time commitment. Respondents noted that patient aggression and violence often accompany medical disputes. In such cases, either the patient or the hospital may seek a solution through legal means such as appeals. Hospitals are required to spend time and money to resolve such issues. In addition, it was sometimes difficult to determine beyond doubt the at-fault party in a medical dispute, and hospitals were consequently required to compensate a patient (and their relatives):

After medical errors, we will certainly compensate patients and their families (…) However, sometimes, it is difficult to determine whether the physician or the patient was at fault in a medical dispute. In such cases, we also have to pay some compensation to patients (B1)

Second, if a physician is injured by a patient and/or their relatives/friends, the hospital will pay compensation to cover the physician’s medical or hospitalisation costs: *“If a physician in our hospital is injured due to a patient’s attack, we will definitely pay their related treatment expenses.”* (B3)

Third, respondents from a secondary hospital claimed that, due to image and reputational damage to their hospital, patients might in the short term choose other hospitals for their treatment, which would mean that the hospital will lose a certain amount of income:

You know, the public will become less inclined to come to secondary hospitals for treatment. After violence occurs, some patients are likely to choose to go to a more prestigious hospital for treatment. (B4)

## Discussion

Studies investigating the consequences of aggression and violence by patients (and their relatives/friends) had a strong focus on the individual level, with little research focused on impacts at the team and organisational levels. As a result, strategies to cope with and prevent such violence also tended to be largely centred on diminishing the individual-level impacts. However, the impact of patient (and their relatives/friends) aggression and violence on teams and hospitals cannot be denied. Based on this study, aggression and violence by patients (and their relatives/friends) can, at the team level, affect team climate, team communication, team belief and team resources. At the hospital level, such aggression and violence can have negative financial impacts (in paying compensation and incurring additional costs) and societal impacts (image and reputational damage and public distrust). Although support from peers, leaders and hospital administrators was identified as an important source for physicians in dealing with violent incidents, often verbal peer support was the only form provided.

### Consequences of an unsafe climate and safety concerns

This study showed the impact of patient (and their relatives/friends) aggression and violence against physicians on the team climate. Consistent with previous research,^9^ this study found that in an unsafe working environment, individual negative emotions can easily permeate the entire team and influence the moods of other team members and, consequently, shape the team atmosphere. This phenomenon aligned with collective emotion theory: team members may react similarly to shared events and therefore experience similar feelings of frustration.^9^ They can also influence each other’s emotions, resulting in a mood convergence.^31 32^ Unpleasant emotions are more likely to lead to emotional contagion than pleasant ones among group members, which in turn can create a negative affective tone within the team.^33^ In addition, group members’ emotions can affect group level outcomes through emotional contagion.^10 33^ Consistent with this argument, as well as other prior research, this study found that the impacts of a negative team climate can extend to team behaviour, such as negatively affecting team communications as well as boosting employee turnover intention.^10^,^14^

Furthermore, patient (and their relatives/friends) aggression and violence did not only have negative consequences for physicians, team functioning and hospital performance, but also for patients themselves. Due to physicians developing a self-protective motivation, this study showed that defensive medical activities may occur, such as making additional unnecessary referrals, opting for only conservative, potentially less optimal, treatments and over-testing; again an observation in line with other studies.^34 35^ These defensive medical practices not only tended to lead to higher healthcare costs but also wasted scarce resources.^36 37^

### Cultural differences in perceptions of violence and support

Existing research suggests that patient aggression and violence can lead to increased perceived and actual workload among healthcare professionals.^38 39^ For instance, a study conducted in Australian hospitals found that workplace violence resulted in task redistribution and employee absence, leading to additional burden on healthcare professionals within the group.^39^ However, our study did not find a similar perception among Chinese physicians, which may be attributed to cultural differences. In China’s collectivist organisational culture, patient aggression and violence could be regarded as shared team concerns rather than as individual responsibilities.^40 41^ Consequently, physicians may tend to view coping with violent incidents as a collective responsibility rather than an additional workload imposed on specific individuals. While respondents acknowledged that the responsibilities of absent colleagues’ (due to injury or distress) had to be redistributed, they did not explicitly regard this as an increased workload but rather as an expected professional duty. Inversely, in more individualistic cultures, workplace responsibilities tend to be perceived as personal rather than shared, resulting in a stronger awareness of individual accountability.^40^ Therefore, experiencing violence could be seen as an individual burden rather than a collective issue within the team, which means that workplace violence is synonymous with an increase in stress and workload in such cultural contexts.^38 39^ However, our study revealed that these violent incidents still affected team resources in other notable ways. In particular, due to staff turnover and absenteeism, these violent occurrences can strain human resources within the team. Material resources were also impacted since medical equipment or facilities could be damaged during violent incidents. Clearly, the Chinese cultural context may shape physicians’ understanding and responses to expressions of patient aggression and violence, while the underlying strain on both human and material resources should not be overlooked.

Moreover, cultural factors may also influence the effectiveness of various forms of support methods in assisting physicians cope with patient aggression and violence. Consistent with previous studies, our research underscored the critical role of both peer support and leadership support in managing patient aggression and violence.^9 42 43^ However, in the context of China’s high power-distance culture,^44^ leadership support appeared as particularly important. Previous studies in China claimed that healthcare professionals often expect more support from leaders than from colleagues or family members.^43 45^ In contrast, Western cultures (ie, low power distance and individualism) and formal support methods (eg, institutional policies) may be more valued than support from leaders and team members.^46^ In addition to this, the effectiveness of peer support was also debated in Western countries.^47^ For instance, some studies suggested that, in the absence of appropriately structured guidance, peer support may lead to emotional contagion that spread within the team and exacerbated psychological distress among team members.^47 48^ However, this aspect has not been emphasised in the Chinese context.^49^ Specifically, in coping with stress, Western cultures tended to focus on seeking personal and independent support, while the collectivist Chinese culture encouraged individuals to rely more on social networks and placed a greater focus on group relationships when seeking help.^49^

### The organisational-level impacts

The finding that aggression and violence by patients and their relatives/friends against physicians affects hospitals financially was consistent with previous studies. Such violence has both direct and indirect costs for the organisation, including absenteeism, resignations, lack of motivation and requirement to pay compensation.^8 39 50^ Patient (and their relatives/friends) aggression and violence also had a societal impact. Related negative media reports can increase patients’ distrust in hospitals and were not conducive to building a good image of healthcare professionals. This could even adversely impact the prevalence of aggression and violence by patients and their relatives/friends. Indeed, previous studies have found that superficial or erroneous reporting of medical disputes in new media outlets is a risk factor for patient aggression and violence.^24 51^

### Strengths, implications and limitations

Through incorporating perspectives at individual, team and organisational levels in the Chinese context, our study contributed to the existing research on the aftermath of patient aggression and violence against physicians. Compared with most previous studies which mainly investigated the negative consequences of workplace violence at individual level (eg, physical injury and burnout),^2 52^ our study further explored the impact of patient aggression and violence on teams and healthcare institutions. Additionally, although our study is in line with existing research on nurse teams, which has also shown the effects of workplace violence on the team level (eg, team efficacy),^53 54^ our study further revealed that team climate, communication and beliefs are affected by patient aggression and violence against physicians. At the organisational level, prior studies have shown that workplace violence resulted in financial losses for hospitals, such as compensation, increased security costs and staff turnover.^8 55^ Building on this, our study found a more nuanced relationship between patient aggression and violence, financial losses (eg, reduced income) and public distrust, especially in Chinese secondary hospitals. Based on that, by integrating insights from multiple levels (individual, team, and organisational), our study provided a comprehensive understanding of the consequences of patient aggression and violence against physicians in Chinese hospitals.

Based on the findings from our study, three main strategies emerge for addressing the negative impacts of patient (and their relatives/friends) aggression and violence in Chinese hospitals. First, recovering the team climate after a violent incident, and building a safe and positive working environment especially requires attention. Here, introducing safety policies and programmes (eg, safety training and the availability of safety-related resources) proved effective in creating safer workplaces.^56^ Second, leaders and peers should provide proactive support to physicians who experience patient (and their relatives/friends) aggression and violence. To improve the effectiveness of support interventions, in addition to the emotional support (eg, verbal comfort) highlighted in our study, a broader range of forms of support, such as paid leave and professional interventions (eg, individual coaching), should be considered to strengthen support interventions. In facilitating support, hospitals should take the possible emotional contagion into account and provide guidance to prevent this. Third, the top-level hospital management should consider how to cope with the negative impact of media reports about workplace violence. For example, hospitals could formulate contingency plans to identify and manage the impact of negative news and could increase their influence through disseminating positive publicity about their hospital.^43^

There are four main limitations that should be taken into account when interpreting the findings and drawing conclusions in this study. First, since respondents were recruited using the authors’ own networks, a selection bias might be present. However, the severity of any selection bias is probably limited as respondents were selected from the broad networks of two of the authors and therefore came from different regions and different levels of hospital, and involved different types of people working in hospitals. However, respondents who preferred not to share their experiences were less likely to have been included in the sample, and this could lead to a bias. Second, respondents may have been reluctant to present the full picture, regardless of the emphasis we placed on anonymity. This reluctance could be explained by a social desirability bias, whereby respondents may have provided responses they perceived as socially acceptable rather than fully disclosing their true experiences. For example, physicians may not have fully disclosed their experiences because of a possible stigma attached to being a victim of violence. Similarly, leaders may also have downplayed certain aspects to protect a hospital’s reputation and image. Third, as our study did not distinguish different types of patient (and their relatives/friends) aggression and violence, specifically verbal and physical violence, the potential for different outcomes resulting from these various types of violence has not been explored. Finally, since patient aggression and violence are highly context-dependent phenomena, the consequences on the team and organisational levels should be placed in a specific national context. This study explained how some findings fit the specific Chinese culture. Consequently, the generalisability of our findings to other cultures and contexts will be limited. Nevertheless, this study can serve as a starting point for similar studies in other countries and cultures.

## Conclusions

This study, by conducting interviews, investigated the impact of patient (and their relatives/friends) aggression and violence against physicians on the team and organisational levels in Chinese hospitals. At the team level, patient aggression and violence can create an unsafe and depressing team climate and damage team resources, leading to decreased communication within the teams and the adoption of low-risk treatments. At the organisational level, patient aggression and violence can have negative financial impacts (ie, involving compensation and additional costs) and societal impacts (ie, image and reputational damage, and public distrust). Recovering a team climate after a violent incident and providing a variety of forms of support can be considered as two important approaches to address these negative impacts of such violence on the team and organisational levels.

## Supplementary material

10.1136/bmjopen-2024-092229 online supplemental file 1

## Data Availability

Data are available upon reasonable request.
