# Supplementary material for: Impact of patient aggression and violence against physicians on the team and organisational levels in China: a qualitative study
Source: BMJ Open. 2025 May 28;15(5):e092229. doi: 10.1136/bmjopen-2024-092229 (PMC12121591; doi:10.1136/bmjopen-2024-092229)
Supplement: online supplemental file 1 [file bmjopen-15-5-s001.docx]

Supplemental Material

Appendix 1: Guideline interviews

| **Participant Type** | **Guideline Interview Questions** |
| --- | --- |
| Physicians | 1. Can you describe a situation in which patient aggression and violence have occurred? 2. How did that experience affect you? Follow up: Did it also affect you physically, emotionally, in terms of your work performance? Were there any other consequences? 3. Could you think of a situation in which you experienced/witnessed patient aggression and violence? How did this incident affect your work in your team? And teamwork as a whole? 4. Follow-up based on previous answers: Given the (negative) impact of the patient aggression and violence both on you and your work in the team, what kind of help did your team and hospital provide you to address these negative effects? 5. When you experienced patient aggression and violence, did you ask your leader for help or report these incidents to your leader? How did your leader respond to you? What roles do leaders have in this situation currently? How would you like the leader to respond to you? 6. When your colleagues (team members) experienced patient aggression and violence, how did she/he react? Did this situation influence you, like asking you for help, or affecting your work in the department due to aggressively approached colleagues? 7. Did you offer help to colleagues who experienced patient violence? What kind of help did you provide? |
| Team leaders | 1. When a member of your team experienced patient aggression or violence, how did they react? How did the rest of the team respond? 2. How did these reactions affect the team? Follow-up questions: 3. If a physician in your team was injured due to patient attacks, did these negative effects influence your team and how? 4. If patient aggression and violence affected the psychological well-being of the physician within your team, did these negative effects influence your team and how? 5. What other effects do you think patient aggression and violence has on the team? 6. When someone in your team experienced patient violence, how do you normally respond as a leader? What do you see as your role in handling these situations? 7. Have you ever personally experienced patient violence as a team leader? If so, do you think it has affected your team? 8. Which tools are most used to cope with the negative effects of patient violence, and which tools are available, like compensating physicians? Is there a need that is not covered by the available interventions/tools? |
| Hospital board members | 1. How does patient aggression and violence impact the hospital? 2. What policies and interventions has the hospital implemented to reduce patient aggression and violence and minimize its negative impact? 3. The team leaders pointed out the impact of patient aggression and violence on the team (based on interviews with team leaders). What solutions does the hospital offer for coping with these effects? Besides that, what other negative effects do you think patient aggression and violence has on the team? 4. What role does the team leader play in these situations? And what about you—what is your role when these incidents happen? |

Appendix 2: Characteristics of participants

| Working profession | Department | Gender | Patient aggression and violence | Hospitals |
| --- | --- | --- | --- | --- |
| Physician (P1) | Gynecology and obstetrics | Female | Experienced and witnessed | Hospital A |
| Physician (P2) | Medical Oncology | Male | Witnessed |  |
| Physician (P3) | Otolaryngology | Female | Experienced and witnessed |  |
| Physician (P4) | Cardiology | Male | Witnessed |  |
| Physician (P5) | Enterosurgery | Male | Witnessed |  |
| Team leader (T1) | Emergency department | Male | Experienced and witnessed |  |
| Team leader (T2) | Medical affairs | Female | Witnessed |  |
| Board member of hospital (B1) |  | Female | Witnessed |  |
| Physician (P6) | Traumatic orthopedics | Female | Witnessed | Hospital B |
| Physician (P7) | Traumatic orthopedics | Male | Witnessed |  |
| Physician (P8) | Orthopedics | Male | Experienced and witnessed |  |
| Physician (P9) | Gynecology and obstetrics | Female | Experienced and witnessed |  |
| Team leader (T3) | Traumatic orthopedics | Male | Experienced and witnessed |  |
| Team leader (T4) | Traumatic orthopedics | Female | Experienced and witnessed |  |
| Board member of hospital (B2) |  | Male | Experienced and witnessed |  |
| Physician (P10) | Acupuncture department | Female | Witnessed | Hospital C |
| Physician (P11) | Psychiatry | Female | Witnessed |  |
| Physician (P12) | Psychiatry | Male | Experienced and witnessed |  |
| Physician (P13) | Internal medicine department | Female | Experienced and witnessed |  |
| Team leader (T5) | Geriatric care | Male | Experienced and witnessed |  |
| Team leader (T6) | Geriatric care | Male | Witnessed |  |
| Board member of hospital (B3) |  | Female | Experienced and witnessed |  |
| Physician (P14) | Dental department | Male | Witnessed | Hospital D |
| Physician (P15) | Dental department | Female | Experienced and witnessed |  |
| Physician (P16) | Ophthalmology department | Male | Experienced and witnessed |  |
| Physician (P17) | Otolaryngology department | Female | Witnessed |  |
| Team leader (T7) | Orthopedics & Physician-patient office | Female | Experienced and witnessed |  |
| Team leader (T8) | Ophthalmology | Male | Experienced and witnessed |  |
| Board member of hospital (B4) |  | Male | Experienced and witnessed |  |
