# Supplementary material for: Impact of patient aggression and violence against physicians on the team and organisational levels in China: a qualitative study
Source: BMJ Open. 2025 May 28;15(5):e092229. doi: 10.1136/bmjopen-2024-092229 (PMC12121591; doi:10.1136/bmjopen-2024-092229)
Supplement: Supplementary file 2 [file bmjopen-2024-092229.reviewer_comments.pdf]

PEER REVIEW HISTORY

BMJ Open publishes all reviews undertaken for accepted manuscripts. Reviewers are asked to complete a checklist review form and are provided with free text boxes to elaborate on their assessment. These free text comments are reproduced below.

ARTICLE DETAILS

Title (Provisional)

The impact of patient aggression and violence against physicians on the team and organizational levels in China: a qualitative study

Authors

WU, YUHAN; Ahaus, Kees; Zhao, Dahai; Buljac-Samardzic, Martina

---

VERSION 1 - REVIEW

**Reviewer**	**1**
**Name**	**Ranjan, Piyush**
**Affiliation**	**All India Institute of Medical Sciences, Medicine**
**Date**	**13-Oct-2024**
**COI**	**None**

The present study aims to explore impact of patient aggression against physicians on a team and organisational level and has been successful in highlighting and bringing forth important and novel insights about the same. The paper was easy to comprehend and interest to read throughout. The qualitative semi-structured interview format allowed for exploration of the physician, team and organisation's perspective, without compromising their subjectivity. This lays a strong groundwork for quantitative research.

However, for all its strengths, it would benefit from a few pointers that are listed below:

1. A table listing out the key and pertinent questions asked would be beneficial to understand how the framing of the questions may have facilitated or interfered with the process of inquiry.
2. The discussion section would benefit from comparing these findings to more individualistic, Western cultures and exploring what possible differences may arise when generalisations are made.

3. Discussion can elaborate on how this study adds to the pool of knowledge regarding this topic. How is this study able to justify the tag of 'a more comprehensive investigation'. This can be mitigated by placing the current findings within the larger context of previous literature, and seeing the contrast to highlight the current study's necessity and strength.

**Reviewer**	**2**
**Name**	**Sarıca Çevik, Hüsna**
**Affiliation**	**Ankara University School of Medicine, Family Medicine**
**Date**	**15-Jan-2025**
**COI**	**None**

I find it very important to deeply investigate the issue of violence against physicians and other healthcare professionals, which is unfortunately increasing in frequency and severity, with qualitative research. This study, with its potential to significantly impact the safety and well-being of physicians, is of great significance. I congratulate the authors for their work. Please see my comments below. /

Introduction:

Although the study investigates the impact of patient

aggression and violence against physicians on the team and organizational levels, and conducted in China, adding information about the situation in other continental countries to the Introduction section might be interesting and recommended. The following references are available: 1) Eshah N, Al Jabri OJ, Aljboor MA, et al. Workplace Violence Against Healthcare Workers: A Literature Review. SAGE Open Nursing. 2024;10. doi:10.1177/23779608241258029 / 2) Yücel Özden KB, Sarıca Çevik H, Asenova R, Ungan M. Guardians of health under fire: Understanding and combating violence against doctors. Aten Primaria. 2024 Sep;56(9):102944. doi: 10.1016/j.aprim.2024.102944. Epub 2024 Apr 27. PMID: 38678853; PMCID: PMC11066614. /

Methods:

China is a large country, and patient sociodemographics vary by region. You mentioned that the hospitals where you conducted in-depth interviews were from different areas. Please explain these areas briefly so the reader can better understand that the results cover various regions of China.

In the manuscript of a study that has already been completed, it is recommended that the language be in the past. For example, it would be good to correct sentences that contain future tense, such as "The intention was to achieve data saturation, and the required

number of interviews will depend on the nature of the topic, the quality of the data, and the method of analysis." Please check throughout the manuscript.

Although you have provided the references, it is recommended that you add and define a few sentences about the qualitative analysis type described by Gioia and Chittipedi.

I could not understand data analysis item 2. Did the researchers independently identify secondary themes and compare their results, and disagreements were resolved through discussion? /

Results:

It is important to highlight the experiences of female participants in your study. Were there any specific situations that female physicians mentioned? This is particularly important when we consider the prevalence of violence in the world, where female physicians unfortunately face a higher risk of assault.

"Support from team members and leaders: valuable but limited in form" - This theme is not understandable at first reading. You may consider changing the wording for "Limited in form". /

Discussion:

"For example, patient aggression and violence increased the workload of other staff in Australian hospitals.31 However, this was not the case in this study, which can be explained by Chinese culture." - This sentence in the discussion contradicts your theme, "Team resources: impacts on material and human resources". /

Limitations:

You have explained the limitations with examples regarding social desirability bias, it would be good to give it as a term as well.

VERSION 1 - AUTHOR RESPONSE

<u>Reviewer(s)' Comments to Author:</u>

Reviewer #1:
1. A table listing out the key and pertinent questions asked would be beneficial to understand how the framing of the questions may have facilitated or interfered with the process of inquiry.

Our answer: Thank you for this observation. We added Appendix 1 to show the interview guidelines.

2. The discussion section would benefit from comparing these findings to more individualistic, Western cultures and exploring what possible differences may arise when generalizations are made.

Our answer: Thank you for this insightful suggestion. We agree that comparing our findings with those from more individualistic, Western cultures can provide valuable context

and enhance the interpretation of our results. Therefore, we revised "4.2 Support in the Chinese context" into "4.2 Cultural differences in perceptions of violence and support" in the Discussion section. We have expanded the discussion to highlight key differences between collectivist and individualist cultures in how patient aggression and violence is perceived and managed, ensuring a more comprehensive interpretation of our results:

"Existing research suggests that patient aggression and violence can lead to increased perceived and actual workload among healthcare professionals.[38 39] For instance, a study conducted in Australian hospitals found that workplace violence resulted in task redistribution and employee absence, leading to additional burdens on healthcare professionals within the group.[39] However, our study did not find a similar perception among Chinese physicians, which may be attributed to cultural differences. In China's collectivist organizational culture, patient aggression and violence could be regarded as shared team concerns rather than as individual responsibilities.[40 41] Consequently, physicians may tend to view coping with violent incidents as a collective responsibility rather than an additional workload imposed on specific individuals. While respondents acknowledged that the responsibilities of absent colleagues' (due to injured or distressed) had to be redistributed, they did not explicitly regard this as an increased workload, but rather as an expected professional duty. Inversely, in more individualistic cultures, workplace responsibilities tend to be perceived as personal rather than shared, resulting in a stronger awareness of individual accountability.[40] Therefore, experiencing violence could be seen as an individual burden rather than a collective issue within the team, which means that workplace violence is synonymous with an increase in stress and workload in such cultural context.[38 39] However, our study revealed that these violent incidents still affected team resources in other notable ways. In particular, due to staff turnover and absenteeism, these violent occurrences can strain on human resources within the team. Material resources were also be impacted, since medical equipment or facilities could be damaged during violent incidents. Clearly, the Chinese cultural context may shape physicians' understanding and responses to expressions of patient aggression and violence, while the underlying strain on both human and material resources should not be overlooked.

Moreover, cultural factors may also influence the effectiveness of various forms of support methods in assisting physicians cope with patient aggression and violence. Consistent with previous studies, our research underscored the critical role of both peer support and leadership support in managing patient aggression and violence.[9 42 43] However, in the context of China's high power-distance culture,[44] leadership support appeared as particularly important. Previous studies in China claimed that healthcare professionals often expect more support from leaders than from colleagues or family members.[43 45] In contrast, Western cultures (i.e., low power distance and individualism), formal support methods (e.g., institutional policies) may be more valued than support from leaders and team members.[46] In addition to this, the effectiveness of peer support was also debated in Western countries.[47] For instance, some studies suggested that, in absence of appropriately structured guidance, peer support may lead to emotional contagion that spread within the team and exacerbated psychological distress among team members.[47 48] However, this aspect has not been emphasized in Chinese context.[49] Specifically, in coping with stress, Western cultures tended to focus on seeking personal and independent support, while the collectivist Chinese culture encouraged individuals to rely more on social networks and placed a greater focus on group relationships when seeking help.[49]"
(Page 20-22)

3. Discussion can elaborate on how this study adds to the pool of knowledge regarding this topic. How is this study able to justify the tag of 'a more comprehensive investigation'. This can be mitigated by placing the current findings within the larger context of previous literature, and seeing the contrast to highlight the current study's necessity and strength.

Our answer: Thank you for this valuable suggestion. We have revised the Discussion section to explicitly highlight how our study adds to the existing pool of knowledge and justifies the label of "a more comprehensive investigation." Specifically, we modified the "Practical implications and Limitation" section into "Strengths, implications and limitations". And we have added a new paragraph in "Implications and Limitation" section that places our findings within the larger context of previous literature and contrasts them with earlier studies to underscore the necessity and strength of our research:

"*Through incorporating perspectives at individual-level, team-level, and organizational-level in the Chinese context, our study contributed to the existing research on the aftermath of patient aggression and violence against physicians. Compared to most previous studies which mainly investigated the negative consequences of workplace violence at individual level (e.g., physical injury and burnout),*[52 53] *our study further explored the impact of patient aggression and violence on teams and healthcare institutions. Additionally, although our study is line with existing research on nurse teams that has also shown the effects of workplace violence on nurse teams (e.g., team efficacy),*[54 55] *our study revealed that team climate, communication, and beliefs are affected by patient aggression and violence against physicians. At the organizational level, prior studies have shown that workplace violence resulted in financial losses for hospitals, such as compensation, increased security costs and staff turnover.*[56 57] *Building on this, our study found a more nuanced relationship between patient aggression and violence, financial losses (e.g., reduced income), and public distrust, especially in Chinese secondary hospitals. Based on that, by integrating insights from multiple levels (individual, team, and organizational), our study provided a comprehensive understanding of the consequences of patient aggression and violence against physicians in Chinese hospitals.* " (Page 23)

Reviewer #2:

Introduction:

1.Although the study investigates the impact of patient aggression and violence against physicians on the team and organizational levels, and conducted in China, adding information about the situation in other continental countries to the Introduction section might be interesting and recommended. The following references are available: 1) Eshah N, Al Jabri OJ, Aljboor MA, et al. Workplace Violence Against Healthcare Workers: A Literature Review. SAGE Open Nursing. 2024;10. doi:10.1177/23779608241258029 / 2) Yücel Özden KB, Sarıca Çevik H, Asenova R, Ungan M. Guardians of health under fire: Understanding and combating violence against doctors. Aten Primaria. 2024 Sep;56(9):102944. doi: 10.1016/j.aprim.2024.102944. Epub 2024 Apr 27. PMID: 38678853; PMCID: PMC11066614.

Our answer: We appreciate this insightful comment regarding the need to include information about patient aggression and violence against physicians in other continental countries. We have revised the Introduction section to incorporate findings from recent studies on workplace violence in healthcare settings beyond China. Specifically, we have added the following paragraph in the Introduction section:

"*A wide range of worldwide studies have highlighted the prevalence of patient aggression and violence and indicated its negative impact on healthcare professionals and institutions. In Europe, physicians in Bulgaria and Spain frequently experience violence from patients and their families, resulting in psychological and work functional impacts.*[15 16] *Belgian and Italian studies have shown sever risk factors associated with patient aggression and violence against healthcare workers, both at the individual and at the systemic level.*[17 18] *Multiple studies in Turkey and India have shown that the prevalence of workplace violence against physicians frequently occurs in emergency departments, leading to occupational stress and burnout.*[19 20 21]

Although aggression and violence in healthcare settings is a global issue, its prevalence and impact vary by culture and healthcare system. [22] *In China, more than 60% of healthcare professional have experienced workplace violence, and particularly from patients and their relatives.*[23] [24] *China's healthcare system also faces persistent challenges that place significant strain on hospitals, increasing the risk of patient aggression and violence. These challenges include high patient expectations, resource limitations, and a preference for patients to directly seek care at higher-level hospitals due to the lack of a primary care gatekeeper system.*[25] [26]" (Page 5)

Methods:

1.China is a large country, and patient sociodemographics vary by region. You mentioned that the hospitals where you conducted in-depth interviews were from different areas. Please explain these areas briefly so the reader can better understand that the results cover various regions of China.

Our answer: Thank you for this suggestion. We acknowledge that China is a vast country with significant regional variations in patient sociodemographics. Therefore, we explained these four different areas in the manuscript:

"To attain a diverse sample, four public hospitals (two secondary and two tertiary) located in eastern China (i.e., Shanghai, Shandong, Jiangsu, and Zhejiang) were selected. These regions were selected for the variety in hospital landscapes. Specifically, Shanghai possesses advanced medical technology and famous hospitals, which attracts many Chinese patients. In contrast, due to urban-rural divide, Shandong faces a range of problems (e.g., distribution of healthcare resources) that impact access to healthcare services. With proactive government policies and a well-developed economy, Jiangsu has well-organized hospital management and physician training programs that set an example for improving healthcare efficiency. Zhejiang is known for the widely recognized hospital' s management model through healthcare reforms and an innovative service model of urban-rural healthcare integration (e.g., digital healthcare). The regions present a comprehensive view of the hospital dynamics in Chinese hospitals. (Page 7)

2.In the manuscript of a study that has already been completed, it is recommended that the language be in the past. For example, it would be good to correct sentences that contain future tense, such as "The intention was to achieve data saturation, and the required number of interviews will depend on the nature of the topic, the quality of the data, and the method of analysis." Please check throughout the manuscript.

Our answer: Thank you for this observation. We changed the tense from future to past. For example: We changed "*The intention was to achieve data saturation, and the required number of interviews will depend on the nature of the topic, the quality of the data, and the method of analysis*." into "*The intention was to achieve data saturation, and the required number of interviews depended on the nature of the topic, the quality of the data, and the method of analysis*"; "*The combination of these three groups will provide a comprehensive view of the impact of patient (and their relatives/friends) aggression and violence on teams and organizations*." into "*The combination of these three groups provided a comprehensive view of the impact of patient (and their relatives/friends) aggression and violence on teams and organizations*."

3. Although you have provided the references, it is recommended that you add and define a few sentences about the qualitative analysis type described by Gioia and Chittipedi.

Our answer: Thank you for this suggestion. We added a few sentences to explain the

qualitative analysis process proposed by Gioia et al.:

"*The qualitative data was analyzed using the method proposed by Gioia et al.,* [28 29 30] *including 1) generating first-order codes (i.e., raw codes directly extracted from the interview transcripts); 2) integrating first-order codes into second-order themes through axial coding; and 3) ultimately refining higher-order theoretical frameworks.*" (Page 9)

4. I could not understand data analysis item 2. Did the researchers independently identify secondary themes and compare their results, and disagreements were resolved through discussion? /

Our answer: Thank you for this observation. We modified the data analysis item 2) to enhance clarity:

"2) *The open codes were then combined, through axial coding, into second-order themes. Each researcher was involved in parts of the first-order codes, analyzing their underlying meanings, and contributed to the development of second-order themes by interpreting the data through their perspectives and expertise. These second-order themes were then compared and any differences discussed until a consensus was reached. 3) Finally, the second-order themes were grouped into integrative dimensions to form a structured and comprehensive framework for understanding the impact of patient aggression and violence at the team and organizational levels.*" (Page 9-10)

Results:

1.It is important to highlight the experiences of female participants in your study. Were there any specific situations that female physicians mentioned? This is particularly important when we consider the prevalence of violence in the world, where female physicians unfortunately face a higher risk of assault.

Our answer: Thank you for this valuable suggestion. We fully acknowledge the importance of examining gender-specific risks and experiences in workplace violence. In our study, we analyzed the data for gender-related differences but did not identify significant or unique patterns in the experiences of female physicians compared to their male counterparts. To ensure transparency, we have included gender-related details in Appendix 2.

Some Chinese studies suggest that male healthcare providers experience more workplace violence than female physicians (Zhu et al., 2018; Lu et al., 2020; Sun et al., 2022). While global literature frequently reports higher risks for female physicians, other studies indicate that male physicians may face greater exposure to workplace violence, especially physical violence (Belayachi et al., 2010; Mirza et al., 2012; Verma et al., 2019; Mohamad et al., 2021). Given our study's findings and the mixed evidence in existing literature, we did not emphasize gender differences in the main discussion. However, we recognized that this remains an important area for further research.

2."Support from team members and leaders: valuable but limited in form" - This theme is not understandable at first reading. You may consider changing the wording for "Limited in form".

Our answer: Thank you for this suggestion. We modified the "*valuable but limited in form*" into "*valuable yet limited*".

Discussion

"For example, patient aggression and violence increased the workload of other staff in Australian hospitals.[31] However, this was not the case in this study, which can be explained by

Chinese culture." - This sentence in the discussion contradicts your theme, "Team resources: impacts on material and human resources".

Our answer: Thank you for your helpful observation regarding the statement about workload in Australian hospitals and its apparent contradiction with the theme "Team resources: impacts on material and human resources." This comment provides an opportunity to clarify how our findings compare with the existing literature, particularly with the situation in Australian hospitals.

The theme "Team resources: impacts on material and human resources" focuses on both the tangible and intangible resources affected by patient aggression and violence at the team level, such as damage to medical equipment and challenges in retaining staff. In contrast, the statement referring to Australian hospitals highlights the perceived increase in actual workload for other staff following violent incidents, such as task redistribution and additional administrative responsibilities. This was not observed in our study, which can be attributed to the collective responsibility embedded in Chinese culture.

In our findings, while team resources were indeed impacted by patient aggression and violence, we did not find evidence suggesting that the perceived workload of other team members increased significantly. This can be explained by the fact that, within the Chinese cultural context, such incidents are often viewed as a shared responsibility, with colleagues stepping in to cover for the affected members without perceiving it as an additional burden. This cultural dynamic helps explain why the impact on workload differs from what has been observed in Australian hospitals, where individual responsibilities may be more emphasized. Therefore, we added a paragraph to clarify this issue:

"Existing research suggests that patient aggression and violence can lead to increased perceived and actual workload among healthcare professionals.[38 39] For instance, a study conducted in Australian hospitals found that workplace violence resulted in task redistribution and employee absence, leading to additional burdens on healthcare professionals within the group.[39] However, our study did not find a similar perception among Chinese physicians, which may be attributed to cultural differences. In China's collectivist organizational culture, patient aggression and violence could be regarded as shared team concerns rather than as individual responsibilities.[40 41] Consequently, physicians may tend to view coping with violent incidents as a collective responsibility rather than an additional workload imposed on specific individuals. While respondents acknowledged that the responsibilities of absent colleagues' (due to injured or distressed) had to be redistributed, they did not explicitly regard this as an increased workload, but rather as an expected professional duty. Inversely, in more individualistic cultures, workplace responsibilities tend to be perceived as personal rather than shared, resulting in a stronger awareness of individual accountability.[40] Therefore, experiencing violence could be seen as an individual burden rather than a collective issue within the team, which means that workplace violence is synonymous with an increase in stress and workload in such cultural context.[38 39] However, our study revealed that these violent incidents still affected team resources in other notable ways. In particular, due to staff turnover and absenteeism, these violent occurrences can strain on human resources within the team. Material resources were also be impacted, since medical equipment or facilities could be damaged during violent incidents. Clearly, the Chinese cultural context may shape physicians' understanding and responses to expressions of patient aggression and violence, while the underlying strain on both human and material resources should not be overlooked.

Moreover, cultural factors may also influence the effectiveness of various forms of support methods in assisting physicians cope with patient aggression and violence. Consistent with previous studies, our research underscored the critical role of both peer support and leadership support in managing patient aggression and violence.[9 42 43] However, in the context of China's high power-distance culture,[44] leadership support appeared as particularly important.

Previous studies in China claimed that healthcare professionals often expect more support from leaders than from colleagues or family members.[43 45] *In contrast, Western cultures (i.e., low power distance and individualism), formal support methods (e.g., institutional policies) may be more valued than support from leaders and team members.*[46] *In addition to this, the effectiveness of peer support was also debated in Western countries.*[47] *For instance, some studies suggested that, in absence of appropriately structured guidance, peer support may lead to emotional contagion that spread within the team and exacerbated psychological distress among team members.*[47 48] *However, this aspect has not been emphasized in Chinese context.*[49] *Specifically, in coping with stress, Western cultures tended to focus on seeking personal and independent support, while the collectivist Chinese culture encouraged individuals to rely more on social networks and placed a greater focus on group relationships when seeking help.* [49]" (Page 20-22)

Limitations

You have explained the limitations with examples regarding social desirability bias, it would be good to give it as a term as well.

Our answer: Thanks for this suggestion. We agreed that this term is highly relevant to our study, as it helps to better conceptualize the potential reluctance of respondents to fully disclose their experiences due to concerns about social acceptability or stigma. We have revised the limitations section to explicitly introduce and define "social desirability bias" as a key factor that may have influenced the responses of participants. Specifically, we have added the following sentence:

"This reluctance could be explained by a social desirability bias, whereby respondents may have provided responses they perceived as socially acceptable rather than fully disclosing their true experiences." (Page 25)

References

Belayachi, J., Berrechid, K., Amlaiky, F., Zekraoui, A., & Abouqal, R. (2010). Violence toward physicians in emergency departments of Morocco: prevalence, predictive factors, and psychological impact. Journal of Occupational Medicine and Toxicology, 5, 27-32.

Lu, L., Dong, M., Wang, S. B., et al. (2020). Prevalence of workplace violence against healthcare professionals in China: A comprehensive meta-analysis of observational surveys. Trauma, Violence, & Abuse, 21(3), 498-509.

Mirza, N., Amjad, A., Bhatti, A., Mirza, F., Shaikh, K., Kiani, J., Yusuf, M., Khan, M., Nazir, M., Assad, Q., Humayun, A., Kiani, I., Amjad, S., & Imam, S. (2012). Violence and abuse faced by junior physicians in the emergency department from patients and their caretakers: A nationwide study from Pakistan. Journal of Emergency Medicine, 42 (6), 727-733.

Mohamad, O., AlKhoury, N., Abdul-Baki, M. N., Alsalkini, M., & Shaaban, R. (2021). Workplace violence toward resident doctors in public hospitals of Syria: prevalence, psychological impact, and prevention strategies: a cross-sectional study. Human Resources for Health, 19 (1), 8.

Sun, L., Zhang, W., Qi, F., & Wang, Y. (2022). Gender differences for the prevalence and risk factors of workplace violence among healthcare professionals in Shandong, China. Frontiers in public health, 10, 873936.

Verma, R., Bhalla, K., Dhaka, R., Agrawal, G., Dhankar, M., Singh, A., & Kumar, G. (2019). Violence against doctor is a threat in india: A study in a tertiary care institution. Indian Journal of Public Health Research and Development, 10 (8), 472-477.

Zhu, L., Li, L., & Lang, J. (2018). Gender differences in workplace violence against physicians of obstetrics and gynecology in China: A questionnaire in the national congress. PloS one, 13(12), e0208693.

VERSION 2 - REVIEW

**Reviewer**	**2**
**Name**	**Sarıca Çevik, Hüsna**
**Affiliation**	**Ankara University School of Medicine, Family Medicine**
**Date**	**13-Apr-2025**
**COI**	

Thank you for your careful work during the revision process and thorough responses to Reviewers' suggestions. I believe these contributions have enhanced the scientific quality of the study. I wish you success in your future endeavors.
